# The Role of Appearance in Peer Interactions for Early Adolescent Cleft Lip and Palate Patients Post-Repair

**DOI:** 10.3390/children12101351

**Published:** 2025-10-08

**Authors:** Junior Tu, Amber Paige McCranie, Muhammad Daiem, Wei-Lung Lin, Pin-Ru Chen, Shih-Heng Chen, Ting-Chen Lu, Pang-Yun Chou, Lun-Jou Lo, Lukas Prantl, Daniel Lonic

**Affiliations:** 1 Department of Plastic and Reconstructive Surgery and Craniofacial Research Center, Chang Gung Memorial Hospital, Chang Gung University, Taoyuan 333, Taiwan; jr_tu@hotmail.com (J.T.); pinru99@gmail.com (P.-R.C.); shihheng@icloud.com (S.-H.C.); tingchenlu@gmail.com (T.-C.L.); lunjoulo@cgmh.org.tw (L.-J.L.); 2 School of Medicine, UT Southwestern Medical Center, Dallas, TX 75390-9003, USA; amber.mccranie@utsouthwestern.edu; 3 CLAPP Hospital, Lahore 54840, Pakistan; muhammad.daiem@medadvisor-operationsmile.org; 4 Department of Family Medicine, Chang Gung Memorial Hospital, Chang Gung University, Taoyuan 333, Taiwan; cheng831052@gmail.com; 5 Centre of Plastic, Aesthetic, Hand and Reconstructive Surgery, University of Regensburg, Franz-Josef-Strauß-Allee, 93053 Regensburg, Germany; lukas.prantl@ukr.de (L.P.); lonic@mclinic.de (D.L.); 6 MCLINIC Interdisciplinary Specialist Centre, Am Schützeneck 8, 81241 Munich, Germany

**Keywords:** cleft lip and palate (CLP), three-dimensional image technique, 3D imaging, adolescent peers, esthetic evaluation, psychosocial impact

## Abstract

**Highlights:**

**What are the main findings?**

- Both non-cleft and cleft participants could distinguish facial differences in 3D images of children with and without cleft lip and palate (CLP).
- While non-cleft participants rated non-cleft images more positively for appearance, no significant difference was found in social interaction ratings between the two groups.

**What are the implications of the main findings?**

- This study suggests that for early adolescents in Taiwan, appearance alone may not be a major factor affecting social interactions for children who have undergone CLP reconstructive surgery.
- The findings imply that other factors, such as pronunciation difficulties or low self-confidence, may be more significant contributors to social difficulties experienced by children with CLP.

**Abstract:**

Background: This study explored how Taiwanese schoolchildren perceive the appearance of their peers with and without cleft lip and palate (CLP) and whether this perception affects social interactions. We specifically focused on early adolescents with surgically repaired CLP to assess the impact of residual craniofacial deformities. Methods: A cross-sectional design was used, analyzing three-dimensional (3D) surface images of twenty patients with repaired CLP and five without. A total of 91 schoolchildren (40 with CLP, 51 without) served as raters. Participants used a Likert scale to rate images on facial appearance and perceived social acceptance. The study also measured the reliability of its questionnaires using Cronbach’s alpha. Results: All participants successfully differentiated between images of children with and without CLP, though non-cleft participants had significantly better distinguishing abilities. Non-cleft raters consistently gave more positive appearance ratings to non-cleft images, a pattern less evident among cleft raters. While differences in awareness and acceptance between the two groups were not statistically significant, over half of all responses regarding social interaction were neutral. The questionnaires demonstrated high reliability, with Cronbach’s alpha values greater than 0.85. Conclusions: Despite the ability to perceive residual craniofacial differences, appearance alone did not significantly affect social interactions for early adolescent children with surgically repaired CLP in Taiwan. This suggests that other factors may play a larger role in social dynamics within this population.

## 1. Introduction

Cleft lip and palate (CLP) is one of the most prevalent congenital craniofacial anomalies [1,2]. The global prevalence is reported to be between 1 in 700 and 1 in 1500 live births [3,4]. Taking care of CLP patients is a multidisciplinary approach: not only of the medical field with surgeons, anesthesiologists, speech therapy, ENT, orthodontists, dentists and psychologists, but also of social workers, teachers, peers and family [5,6,7,8]. While surgical treatment of the malformation was the primary focus in the earlier days, current concepts also emphasize the patient’s and parent’s mental wellbeing and social integration [9,10,11]. Nevertheless, the social stigma of CLP malformations are high, and patients often remain on the outskirts of society in many regions of the world, especially in developing countries [12,13,14,15,16]. The anatomical malformations can lead to growth impediments, hearing difficulties and deficiencies in oral function and dental health, as well as delays in language development [5,17,18,19,20]. Advances in multidisciplinary teamwork and reconstructive strategies have significantly reduced both functional and esthetic concerns for CLP patients [18,19]. However, the social and psychological impact on children with CLP is a current issue of focus and warrants our attention [21,22].

The main features of CLP malformations are facial asymmetry, underprojection of the midface and nose [5], dental anomalies [23] and dynamic asymmetries related to the smile. Also, growth restrictions due to surgical scarring and anatomical variances lead to unbalanced facial features such as a reduced nasolabial angle, and nasal and upper lip deformities that need to be addressed by surgery and orthodontics [24]. Additionally, subsequent speech problems and negative social experiences in early childhood can lead to social anxiety, mandating speech therapy and psychological support as important cornerstones of comprehensive cleft care [25].

Several studies have indicated that children with CLP between the ages of six and twelve frequently experience social and behavioral inhibition issues [26,27,28]. Observational studies have also demonstrated that pronunciation disabilities in children with CLP can lead to a decline in social acceptance by their peers [29,30,31]. However, there seems to be an inconsistency regarding whether facial discrepancies in CLP children may evoke negative attitudes among peers. Some emphasize that children who look different are more likely to be ignored and rejected [32,33,34]. On the contrary, in Feragen et al.’s studies, there was no significant difference found between reports of peer harassment among those with and without CLP [35,36].

The recently introduced three-dimensional (3D) surface imaging technology has emerged as a major tool for assessing facial deformities, with the advantages of rapid, accurate and non-invasive facial imaging with realistic rendering, dynamic capabilities and reliability for use in various applications [37,38,39,40,41]. This technique can also protect the privacy of vulnerable patient populations, such as those with CLP. In the last decade there has been a massive shift from 2D towards 3D imaging, and the impact and importance of 3D imaging as a basis for CLP surgical planning, documentation and treatment quality control assessment cannot be overstated [42,43]. The potential for standardization and accuracy of digital imaging has risen dramatically, and through the combination of 3D imaging and 3D printing, more standardized approaches in cleft lip and palate procedures and orthognathic surgery are possible, with significantly more accurate reproducible results [44].

To the best of our knowledge, no studies have been performed to date that have utilized 3D imaging in conjunction with peer questionnaires to investigate the differences in esthetic evaluation and interpersonal attitudes towards individuals with CLP compared to typical individuals. The present study aimed to utilize the 3D surface imaging technology to discern the disparity in perception towards patients with CLP among peers, as well as to understand the level of peer awareness and acceptance of children with craniofacial abnormalities post-repair within social contexts. With time, the peer group becomes the most important projection point in terms of developing social skills, social values and self-awareness and self-worth. A large part comes from how children feel perceived by their peers [45,46]. Therefore, this study focuses on the question of how CLP patients perceive themselves and unaffected peers, and vice versa, to investigate the dynamics of inter-peer relationships and help to improve the coexistence and interaction between CLP patients and their environment. Hypothetically, the perception of unaffected peers could be negative in the esthetic field, but it is not clear if the perception translates into social disadvantages for CLP patients.

## 2. Methods

### 2.1. Participants

**Figure 1:**
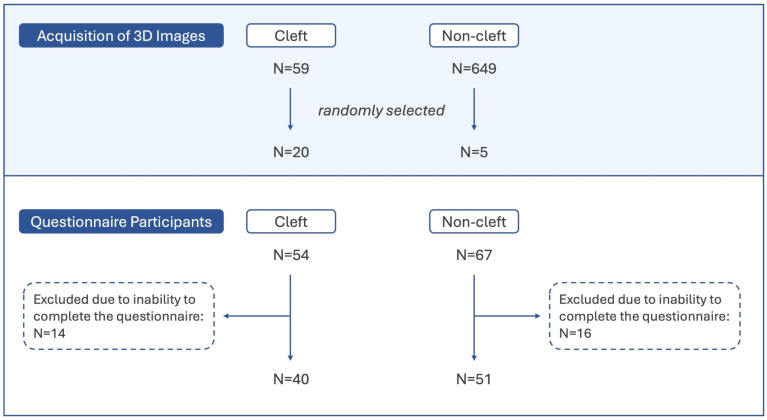
Study population and protocol.

This cross-sectional study was approved by the Institutional Review Board (IRB ID: 202200698B0) of Chang Gung Memorial Hospital and has been performed in accordance with the Declaration of Helsinki, following international guidelines. Parents and/or guardians provided written informed consent, and children verbal consent, for their participation in this study. We randomly invited CLP and non-cleft participants aged 10–12 to complete a questionnaire interview to evaluate 25 3D images. CLP participants were sourced from individuals who had undergone surgical treatment and received regular follow-ups in our craniofacial center; non-cleft participants were randomly recruited from various elementary schools in the Guishan district of Taoyuan City, Taiwan. Written informed consent was obtained from the participants for publication. Participants that were unable to complete the questionnaire due to difficulties during the response process, or due to visual, auditory, neurological and cognitive impairments were excluded. Inclusion criteria for CLP participants were as follows: 1. cleft lip and palate malformation, 2. serial staged reconstruction (lip/nose repair at 3 months of age, palatoplasty at 9–12 months of age, and alveolar bone grafting at 9–12 years of age), 3. age bracket of 9–12 years old and 4. absence of other craniofacial malformations. Exclusion criteria for CLP participants were as follows: 1. manifestation of other craniofacial malformations, 2. aged less than 9 and over 12 years of age, 3. no staged serial reconstruction, as mentioned before, and 4. absence of consent for participation from parents/caregivers and/or patient. Inclusion criteria for the non-cleft participants were 1. absence of craniofacial deformities on themselves or in the family, 2. age bracket of 9–12 years old, 3. consent of parents/caregivers and control individual. Exclusion criteria for the non-affected participants were 1. manifestation of CLP or any other craniofacial malformations, 2. aged less than 10 and over 12 years of age and 3. absence of consent for participation of parents/caregivers and/or patient. Individuals of both groups who were unable to complete the questionnaire due to difficulties during the response process or due to visual, auditory, neurological and cognitive impairments were excluded. After this selection process, finally 40 CLP participants and 51 non-cleft participants, all between the ages of 10 and 12, were included (Figure 1).

### 2.2. Acquisition of 3D Images

**Figure 2:**
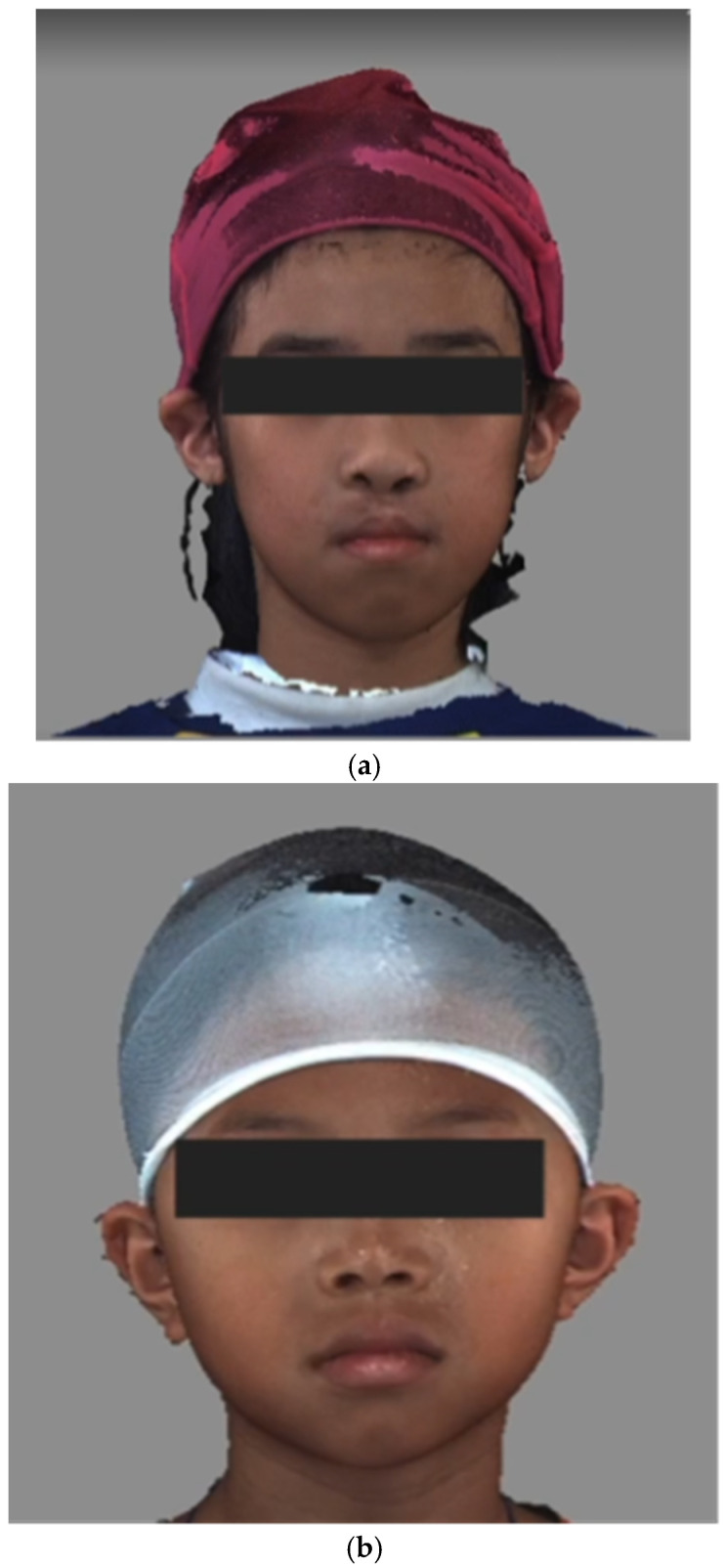
Example of static front view of (**a**) cleft images and (**b**) non-cleft images.

The 3dMD system (3dMD LLC, Atlanta, GA, USA) employs stereophotogrammetry to create three-dimensional surface images. This system was utilized to acquire the images of early adolescent children with (Figure 2a) and without CLP (Figure 2b). The pictures were taken by trained operators under standardized conditions and anonymized to protect the identity of the participants. Written and informed consent for publication of the pictures was obtained by the parents/caregivers as well as verbal consent from the participants. In our center, we follow a standardized protocol of conducting the lip/nose repair at 3–6 months of age, palatoplasty at 9–12 months of age and alveolar bone grafting at 9–12 years of age. The pictures used in this study were taken three to six months after the alveolar bone grafting.

The conditions were standardized to improve the precision of the images, including a natural head position, fixed ambient lighting, neutral facial expressions and the use of a nylon cap to cover the hair (Figure 2a,b, Supplementary Video S1 and Supplementary Video S2) [47,48].

Images of participants with and without craniofacial anomalies were extracted from the database at the Chang Gung Craniofacial Research Center (Taoyuan, Taiwan). The CLP patients had undergone surgical treatment at Chang Gung and appeared for regular follow-up. The database contains photos of 59 CLP patients, taken before and after alveolar bone graft surgery at a six-month interval, from 2017 to 2020. All participants received surgery from one experienced surgeon (LJL). As for non-cleft participants, over 600 3D images in the database were sourced from a previous study conducted between 2016 and 2018, which included healthy pediatric individuals from six to twelve years of age [49]. Written informed consent was obtained from the participants for publication of this study and accompanying images.

Considering the limited patience of early adolescent children, a total of only 25 images were included in the questionnaire, 20 CLP patient 3D pictures and 5 non-cleft individual 3D pictures. (Table 1).

The principal image selection criterion was to ensure integrity in both vertical and horizontal image projections in order to reduce questionnaire evaluation bias caused by image artifacts, such as partial loss, excessive protrusion, and so on. From the database, 20 images of CLP patients and five images of non-cleft children from the ages of nine to 12 were randomly selected for design of the research questionnaire (Figure 1). To enhance the reliability of the assessment, the images of two patients obtained at different time points (all within the nine to 12-year-old age range) were repeated in the questionnaire to verify whether participants filled in their answers seriously.

### 2.3. Questionnaires

The questionnaire comprised images depicting 25 children, including 20 children with CLP and 5 children without any craniofacial anomalies. The sequence of these images was randomized, and each image was presented on a 15 inch laptop monitor, in both dynamic 360-degree rotation and static front view, for a duration of six seconds each [50]. Every participant was required to evaluate each image and answer the four questions designed on a Likert scale of one to five [51].

The four questions on this questionnaire were as follows:

1. Does his/her face appear unusual to you?

2. Would you like to have a chat with him/her?

3. Would you like to make friends with him/her?

4. Does his/her face appear good-looking to you?

All questions were measured using a 5-point Linkert scale:

(5 pt) Very, (4 pt) Somewhat, (3 pt) Neutral, (2 pt) Not really, (1 pt) Not at all.

Although the questions have not been validated in previous studies, they have been designed to fit the target audience of 10–12-year-old schoolchildren, comprising CLP and non-CLP individuals. The questions are short and easy to understand, and could be answered quickly and straight forwardly, minimizing the fatigue of the participants while receiving quick and reliable feedback. The questions have been designed based on two principles being esthetic evaluation and interpersonal attitudes/social behaviors.

Esthetic evaluation questions: “Does his/her face appear unusual to you?” and “Does his/her face appear good-looking to you?”.

Interpersonal Attitudes/Social Behavior questions: “Would you like to have a chat with him/her?” and “Would you like to make friends with him/her?”

Each participant underwent the test individually and received identical instructions from the same staff member prior to the test. After completing the questionnaires, all data were stratified according to the participants’ condition (CLP children and non-cleft children), and further analysis was conducted.

### 2.4. Statistical Analysis

R (4.1.3) was utilized for the statistical analysis. All questionnaire results were sorted through R and visualized into bar graphs. The Item Response Theory Graded Response Model was employed to estimate each participant’s latent capability (θ) in the test, and an independent *t*-test was used to analyze the distribution of each participant’s θ value. The Graded Response Model is a powerful tool in psychometrics, particularly in fields like psychology, education and health research. Here, it is used for the following. Scale development: validating and refining scales that use ordered response formats, like surveys measuring attitudes, personality or self-concept. Statistical significance was defined as a *p*-value of less than 0.05. Cronbach’s alpha was also used to calculate the reliability of each of the four questions. A threshold of acceptability for an alpha value was referred to as greater than 0.7 [52].

## 3. Results

The questionnaire answers from the 40 CLP participants and 51 non-cleft participants, all between the ages of 10 and 12, distributed as follows in Table 2.

**Figure 3:**
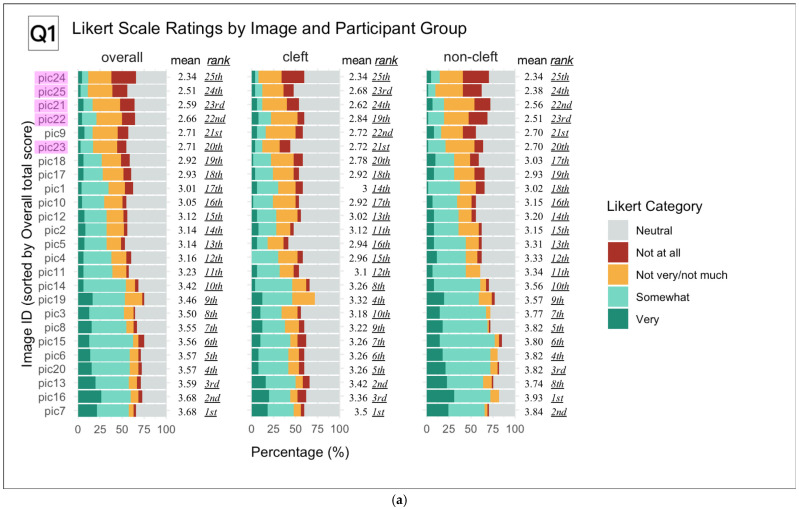

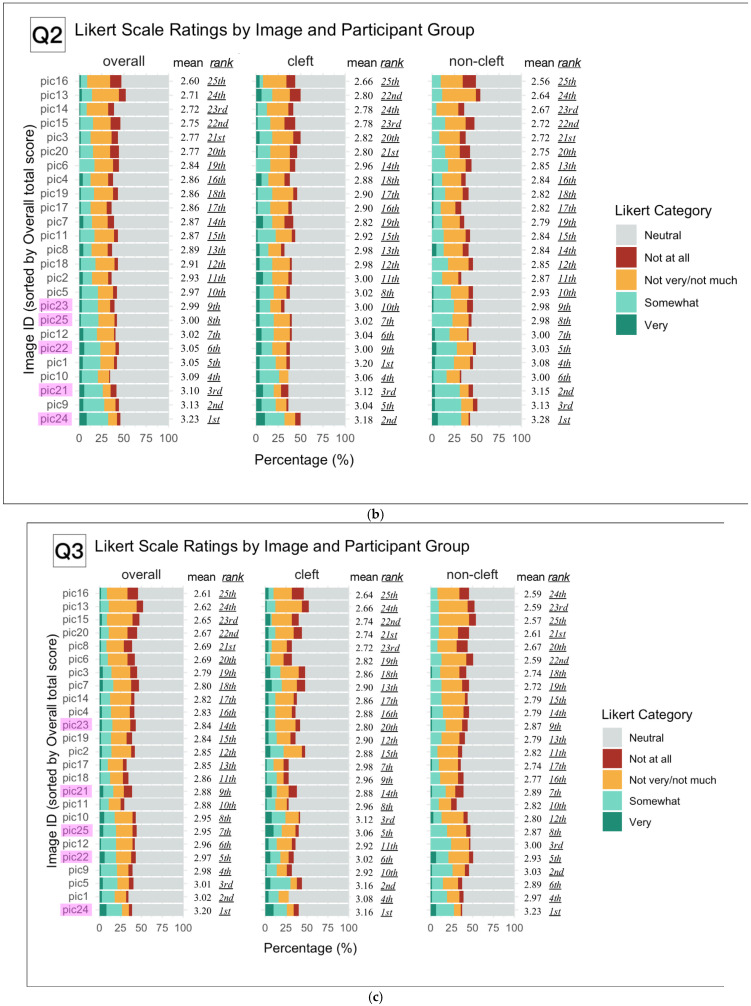

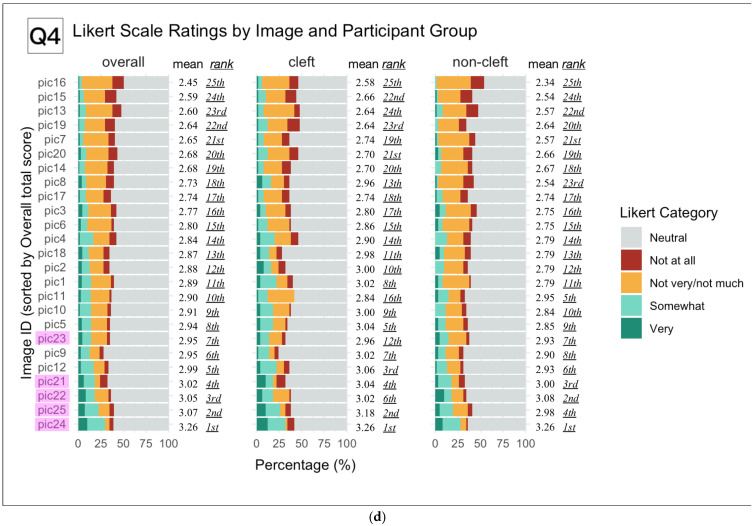
(**a**). Likert Scale Response Distributions for Each Image by Participant Group (Q1) Stacked bar charts display the percentage distribution of Likert scale ratings for each of the 25 images, sorted by overall mean score (left panel). Results are shown separately for the overall cohort, cleft group, and non-cleft group. For each image, the mean Likert score and its rank (lowest to highest) within each group are indicated. Colors represent Likert response categories from “Not at all” to “Very.” Images identified as non-cleft are highlighted in purple. This visualization allows direct comparison of group-specific perceptions for each image and identifies which images were most and least strongly rated across groups. Cleft images showed consistently higher ratings by both groups of participants, overall and respectively. (**b**). Overall and in the non-cleft participant group, consistently higher scores were seen in non-cleft images. This finding was not observed in the cleft participant group. (**c**). Similar result distribution to those in question two. (**d**). Overall and in the non-cleft participant group, higher points were consistently observed for non-cleft images.

The results visualized by the bar graphs revealed a discrepancy in response trends between cleft and non-cleft images, as observed by the two participant groups. In order to differentiate between the image grouping, non-cleft images were highlighted in red (Figure 3a–d).

For question one, “Do you think his/her face looks unusual?”, cleft images were consistently rated higher than non-cleft images among both groups of participants, overall and respectively (Figure 3a).

However, upon further investigation, some cleft images had scores comparable to non-cleft images in the cleft participant group, showing slightly less bias towards a particular set of images. For question two, “Would you like to chat with him/her?”, non-cleft images were more frequently rated with higher scores both by participants overall and non-cleft participants (Figure 3b).

However, among participants in the CLP group, there was a lack of consistency in their ratings of cleft and non-cleft images. As for question three, “Would you like to make friends with him/her?”, some cleft images were rated more positively than in question two, even among the non-cleft participant group (Figure 3c).

The same broad distribution of both cleft and non-cleft image scores seen in the previous question was again observed in the CLP group. In questions two and three regarding social interaction, over 50% of the responses for most images were “Neutral” in both the cleft and non-cleft participant group. Lastly, for question four, “Does his/her face appear good-looking to you?”, participants overall and those among the non-cleft participant group demonstrated a clear consistency in rating non-cleft images with higher scores (Figure 3d).

However, this trend was once again less visible among the CLP participant group; instead, non-cleft image scores were interspersed with those of cleft images, with no particular deviation towards one group or the other. This diffuse pattern was more prominent in the CLP participant group than the non-cleft participant group for questions two, three and four.

**Figure 4:**
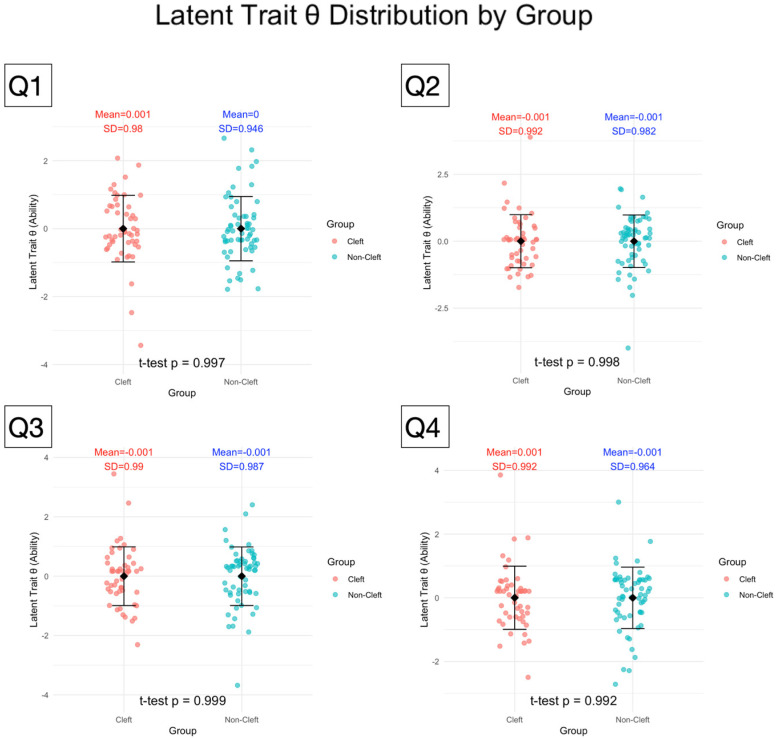
Latent Trait θ Distributions for Cleft and Non-Cleft Groups Across Four Questionnaires Sets.

Using the Graded Response Model, the latent ability of individual participants to distinguish between cleft and non-cleft images within the same question was estimated and quantified as theta (θ) (Figure 4). The normality of (θ) distributions for both cleft and non-cleft groups was assessed with Shapiro–Wilk tests, revealing statistically significant departures from normality across all four questionnaire sets (all *p* < 0.05). Despite this, Levene’s tests confirmed the homogeneity of variances between groups (all *p* > 0.9). Welch’s *t*-tests showed no significant differences in mean θ between cleft and non-cleft participants in any questionnaire (all *p* > 0.99), with similar means and standard deviations across groups. These results indicate comparable latent ability to discriminate cleft images between groups, supporting the robustness of group comparisons despite minor normality violations.

Strip charts display the distribution of latent trait θ estimates, derived via the Item Response Theory Graded Response Model, for cleft and non-cleft participant groups across four questionnaire sets. Each θ value represents a participant’s underlying ability to differentiate cleft images from non-cleft images, based on their pattern of Likert-scale responses to 25 image items in each test. Higher θ values indicate greater capability to discriminate cleft from non-cleft images, while lower values reflect reduced discriminatory ability. Group means and standard deviations are annotated, with statistical testing (*t*-test) revealing no significant difference in latent trait θ between groups across all four tests.

The reliability testing of all four questions, assessed using Cronbach’s alpha values [52], revealed predominantly good to excellent reliability (α ≥ 0.8) across all four questions answered by both non-cleft and cleft participants. However, there were concerns regarding the reliability of question one for participants overall and of questions one and four for the non-cleft participant group viewing non-cleft images, as the alpha values in these categories were less than 0.7 (Table 3).

## 4. Discussion

This is the first study to analyze the differences in perception among peers of patients with CLP using three-dimensional imaging, as well as to assess the level of awareness and acceptance among peers towards children with craniofacial anomalies. Despite having residual deformities or scarring following reconstructive surgery, this study found no significant difference in adolescent responses to questions of social interaction with CLP children in Taiwan.

Previous studies have indicated that children with CLP often experience social and behavioral inhibition issues between the ages of six and twelve [26,27,28]. According to observational research focused on peer interactions, similar findings have been observed in school-age children with CLP; compared to typical individuals, children with CLP spend more time alone, engage less in group play, and experience more negative peer interactions [10,53]. These conditions may stem from the fact that children with CLP often feel frustrated with their appearance [21,25]. Additionally, compared to non-cleft children, those with CLP are more likely to experience speech difficulties [54]. These pronunciation disabilities often result in low self-esteem and negative reactions from peers, thereby affecting their interpersonal relationships [29,30,31,32,33]. Furthermore, Kapp-Simon pointed out that the ages of 10 to 19 are crucial for young adolescents, as they gradually shift their attention from family to peers [55]. Gallagher also emphasized that being able to establish and maintain positive peer relationships is a particularly critical aspect of social competence [56]. Based on current research findings, it has been found that positive peer relationships can enhance social skills among individuals, and vice versa. Therefore, in order to reduce the psychosocial obstacles for patients with CLP, it is important to understand whether facial abnormalities affect peer attitudes.

This study found that both cleft and non-cleft participants were capable of discerning differences in facial features in cleft and non-cleft subject images in question one, pertaining to esthetic evaluation. Post-operative scarring and residual deformities in the cleft images were assumed to be the reasons for these results. This finding could also be explained by a study that found that pre-adolescent observers may judge normal individuals as having similar or even more facial asymmetry when compared with adult raters [57]. However, even though peers were able to distinguish facial discrepancies in this study, the results seen in questions two and three did not show a significant difference between the latent capability (θ) distribution of the cleft and non-cleft groups. In questions of interpersonal attitudes and behavior (two and three), non-cleft participants tended to score non-cleft images more positively, but cleft participants did not demonstrate such bias towards a particular image group. These results can be observed in Figure 3b,c. Additionally, over 50% of the images were scored as neutral by all participants, bringing the overall scoring of each image to a more central position on the bar graphs, with less deviation towards one particular group. Considering the conflicting nature of these findings, this study was unable to conclude with the presence of a significant difference between cleft and non-cleft participants’ attitudes toward children with CLP in the early adolescent stage. These results could also be explained by a lack of information pertaining to personality traits that may affect the child’s decision to have a conversation or develop a friendship with the subject in the image. Both of these theories suggest that the tendency for social inhibition in children with CLP may not be attributed to facial discrepancies alone, but rather the source of such psychosocial issues may be more likely due to low self-confidence, secondary to their appearance satisfaction and pronunciation disabilities [21,31,33]. In addition to this assumption, puzzled and curious reactions from peers may be interpreted negatively by children who are psychologically vulnerable, such as children with CLP, as was observed in the Feragen et al. studies [35,36]. Following the conclusion of this study, non-cleft participants were enrolled in a three-year health education program related to the disorder of CLP. The program may cultivate their empathy and positive attitudes toward CLP children.

The results seen in question four, in which early adolescent peers tended to express more neutral responses regarding subjective esthetic evaluation, as opposed to the more consistently skewed responses seen in question one, requiring comparison to each individual child’s standard of normalcy, may indicate that the ability to identify unfamiliar features may develop earlier than the concept of attractiveness in the course of cognitive development [58,59,60]. Furthermore, there is a stark contrast in participants identifying subjects in the images as appearing “unusual” in question one, but then proceeding to rate the same subjects neutrally or even positively in the subsequent questions two and three, concerning conversation and friendship. This finding could support the assumption presented previously that appearance alone was not a meaningful factor in early adolescents’ desire for social interaction.

Cleft participants showed good reliability in all four questions while non-cleft participants showed questionable and even poor reliability on questions one and four, respectively. We hypothesize that the lower reliability observed in these two questions may be due to the inadequate number of non-cleft images (only five out of the twenty-five total images) [61,62].

There were three limitations within the design of this study that should be addressed by future research. First, all participants were Asian, given that the study recruited exclusively from Taiwan, which may limit the generalizability of the results to other racial or ethnic groups. Furthermore, participants were provided with both static and dynamic three-dimensional craniofacial images with good authenticity; however, whether the results obtained through viewing these images truly represent face to face interpersonal attitudes and behaviors remains debatable. Finally, some non-cleft participants still complained about there being too many images when completing the questionnaire, despite the total number of images having been reduced. Whether the number of images and such complaints will greatly affect the reliability of the questionnaire requires further investigation.

Another structural limitation may be attributed to the statistical power of the study. Due to the nature of the subject with a relatively low global incidence of 1 in 700 to 1500 live births, even comparative studies cannot reveal the full scope of the societal perception of the CLP malformation in a certain age group. Also, in our experience conducting studies with CLP patients, one of the biggest challenges is designing studies with strong statistical power due to the low incidence of CLP malformations, despite the fact that they are one of the most common craniofacial malformations. In addition, extracting a homogenous sample with serial staged reconstruction (lip/nose repair at 3–6 months of age, palatoplasty at 9–12 months of age and alveolar bone grafting at 9–12 years of age) poses another challenge. This, however, is the standard procedure in our craniofacial center and ensured this kind of homogeneity. Therefore, we tried to enroll as many patients as possible in the CLP group and matched them with a similar sized group of non-affected individuals. Subsequently, a sample size calculation was not feasible for this study.

In this study, three-dimensional photography was employed as an esthetic evaluation tool to assess facial deformity. There are many advantages to utilizing three-dimensional imaging; it is minimally invasive, non-radioactive, has quick capture speed and can easily archive images for subsequent analysis [47]. Additionally, three-dimensional images can protect the privacy of cleft patients and eliminate the risk of discrimination. Using this method for a comparative study should enable the participants to base their conscious and unconscious decision making on a much broader information basis by adding the third dimension to the rated data [44]. Lastly, compared with two-dimensions, three-dimensional images expand multi-angle surface coverage, which has been proven to have a high degree of precision and accuracy in several studies [63,64]. We hope this study can provide a benchmark for future research with extensive applications in clinical practice.

Future directions of research may include the development of strategies to raise societal awareness and acceptance for CLP patients, especially in the sensible (pre-) pubertal age group between 10 and 12 years of age. Also, further studies should be conducted regarding the perception of subgroups of CLP and gender-related perception in CLP and non-CLP groups to obtain a more granular view of the interpersonal dynamics in this sensible topic. We are preparing a follow up study to further validate the questionnaire as a tool for assessing peer perception.

## 5. Conclusions

The present study reveals that despite the ability to discern variation in facial features, both cleft and non-cleft participants showed no significant differences in attitudes and behavior when viewing the three-dimensional images. This suggests that craniofacial deformities alone may not greatly affect peer social interactions for children with CLP during early adolescence in Taiwan. This should be important information for the psychological support of CLP patients to strengthen their confidence in social interactions with their peers. Further research should address strategies to improve social interactions in this peer group based on this information.

## 6. Key Points and Relevance

1. This study used three-dimensional imaging to analyze adolescent peer perceptions of patients with cleft lip and palate (CLP).

2. Residual deformities or scarring after reconstructive surgery could still allow for discernible differences between CLP and non-cleft cases when viewed through 3D images.

3. Despite the slight visible differences, both cleft and non-cleft participants did not show significant differences in attitudes or behaviors towards peers with facial features.

4. The findings suggested that craniofacial deformities do not significantly affect peer social interactions for children with CLP in early adolescence in Taiwan.

## Figures and Tables

**Table 1:** Table of 3D images and Subjects characteristics.

	*N*	Male (%)	Age
3D images (*n* = 25)			
Non-clefts	5	2	9.8 (9–12)
Clefts	20	12	9 (9)
Subjects (*n* = 91)			
Non-clefts	51	31	11.33 (10–12)
Clefts	40	25	10.58 (10–12)

**Table 2:** Questionnaire distribution of non-clefts and clefts.

	Non-Cleft Participants (*n* = 51)	Cleft Participants (*n* = 40)
Cleft pictures (*n* = 20)		
Question 1		
N		
Not at all	42	46
Not very/not much	118	148
Neutral	309	355
Somewhat	400	188
Very	151	63
Mean (Median)	3.49 (4)	3.09 (3)
Question 2		
N		
Not at all	66	49
Not very/not much	222	136
Neutral	574	466
Somewhat	145	111
Very	13	38
Mean (Median)	2.82 (3)	2.94 (3)
Question 3		
N		
Not at all	77	53
Not very/not much	239	134
Neutral	563	500
Somewhat	133	73
Very	8	40
Mean (Median)	2.76 (3)	2.89 (3)
Question 4		
N		
Not at all	76	53
Not very/not much	238	129
Neutral	619	504
Somewhat	65	86
Very	22	28
Mean (Median)	2.72 (3)	2.88 (3)
Non-cleft pictures (*n* = 5)		
Question 1		
N		
Not at all	49	23
Not very/not much	78	55
Neutral	78	98
Somewhat	40	16
Very	10	8
Mean (Median)	2.55 (3)	2.66 (3)
Question 2		
N		
Not at all	11	6
Not very/not much	39	28
Neutral	137	124
Somewhat	60	28
Very	8	14
Mean (Median)	3.06 (3)	3.08 (3)
Question 3		
N		
Not at all	16	10
Not very/not much	43	28
Neutral	144	126
Somewhat	43	21
Very	9	15
Mean (Median)	2.95 (3)	3.02 (3)
Question 4		
N		
Not at all	11	11
Not very/not much	35	16
Neutral	162	133
Somewhat	31	25
Very	16	15
Mean (Median)	3.02 (3)	3.09 (3)

**Table 3:** Reliability estimates (Cronbach’s alpha *) and 95% confidence intervals for discriminating cleft and non-cleft images across four questionnaire sets and participant groups. Values indicate the internal consistency of Likert ratings for cleft and non-cleft image items in the overall, cleft, and non-cleft subgroups.

	Q1		Q2		Q3		Q4	
	Cleft	Non-Cleft	Cleft	Non-Cleft	Cleft	Non-Cleft	Cleft	Non-Cleft
Overall	0.876 (0.840–0.907)	0.730 (0.642–0.802)	0.936 (0.918–0.952)	0.838 (0.785–0.881)	0.946 (0.931–0.960)	0.819 (0.759–0.867)	0.929 (0.908–0.947)	0.743 (0.658–0.811)
Non-cleft	0.810 (0.734–0.873)	0.664 (0.510–0.781)	0.931 (0.903–0.954)	0.817 (0.733–0.881)	0.945 (0.923–0.963)	0.814 (0.729–0.879)	0.889 (0.844–0.925)	0.643 (0.478–0.767)
Cleft	0.905 (0.863–0.940)	0.801 (0.698–0.876)	0.941 (0.915–0.962)	0.860 (0.787–0.913)	0.947 (0.923–0.963)	0.826 (0.737–0.892)	0.955 (0.934–0.971)	0.818 (0.723–0.887)

* alpha ≥ 0.9, excellent; 0.9 > alpha ≥ 0.8, good; 0.8 > alpha ≥ 0.7, acceptable; 0.7 > alpha ≥ 0.6, questionable; 0.6 > alpha ≥ 0.5, poor; 0.5 > alpha, unacceptable.

## Data Availability

The datasets used and/or analyzed during the current study are available from the corresponding author on reasonable request.

## Supplementary Materials

The following supporting information can be downloaded at: https://www.mdpi.com/article/10.3390/children12101351/s1, Video S1: The video of patient with cleft. Video S2: The video of patient with non-cleft.

## Abbreviations

The following abbreviations are used in this manuscript:

CLP	Cleft Lip and Palate
